# Correlations between Wastewater Concentrations of Influenza A and Respiratory Syncytial Viruses and Clinical Laboratory Testing and Hospitalization Data, California, USA

**DOI:** 10.3201/eid3213.260306

**Published:** 2026-08

**Authors:** Elisabeth Burnor, Madhura S. Rane, Marisa A.P. Donnelly, Lauren A. White, Cora Hoover, Monica Sun, Caroline Collins, Andrew Abram, Mariah Otstot, Joel Rojas, Cody Berkobien, Seema Jain, Duc J. Vugia, Wayne T.A. Enanoria, Linlin Li, Chad Below, Marlene Wolfe, Alexandria B. Boehm, Tomás M. León, Erin L. Murray, Alexander T. Yu

**Affiliations:** California Department of Public Health, Richmond, California, USA (E. Burnor, M.S. Rane, M.A.P. Donnelly, L.A. White, C. Hoover, M. Sun, C. Collins, A. Abram, M. Otstot, J. Rojas, C. Berkobien, S. Jain, D.J. Vugia, T.M. León, E.L. Murray, A.T. Yu); County of Santa Clara Public Health Department, San Jose, California, USA (W.T.A. Enanoria, L. Li); San Mateo County Health, San Mateo, California, USA (C. Below); Rollins School of Public Health, Emory University, Atlanta, Georgia, USA (M. Wolfe); Stanford University, Stanford, California, USA (A.B. Boehm)

**Keywords:** Influenza, viruses, respiratory infections, wastewater-based epidemiological monitoring, respiratory syncytial virus, RSV, California, United States

## Abstract

We evaluated correlations between influenza A virus and respiratory syncytial virus (RSV) wastewater concentrations in California, USA, and 3 clinical laboratory–based disease surveillance datasets: sentinel laboratory surveillance, mandatory electronic laboratory reporting data, and laboratory-confirmed influenza hospitalizations. We evaluated data from 10 counties and 18 wastewater treatment plants in California during July 2022–March 2024. We observed strong, positive, and statistically significant correlations between individual sewershed-level wastewater concentrations and county-aggregated wastewater concentrations of influenza A and RSV and clinical laboratory–based surveillance datasets (median Kendall τ 0.62 [range 0.40–0.78]; p<0.05). A lead–lag analysis did not show consistent evidence of wastewater leading other surveillance datasets across all counties and wastewater treatment plants (influenza A –16 to 22 days; RSV –8 to 35 days). Wastewater surveillance can augment other disease surveillance modalities and improve situational awareness of influenza A and RSV transmission in communities.

Influenza virus and respiratory syncytial virus (RSV) cause substantial illness and death in the United States (*1*,*2*). Public health agencies use influenza and RSV surveillance data to track annual seasonal patterns, known as respiratory virus seasons. That surveillance is used for hospital capacity planning, public and healthcare provider awareness, resource allocation, and planning for vaccinations and therapeutics. National influenza and RSV surveillance relies primarily on data from state, tribal, local, and territorial health departments. Those entities, in turn, rely on surveillance drawn from heterogenous data sources, including public health laboratories, clinical laboratories, vital statistics offices, health care providers, emergency departments, and long-term care facilities that variably contribute testing, genomic sequencing, and syndromic, hospitalization, and death data (*3*). Although effective, that surveillance approach has heterogenous coverage gaps and is subject to biases caused by disparate healthcare access and healthcare seeking behavior because persons with mildly symptomatic or asymptomatic infections might not seek clinical testing (*4*).

Wastewater surveillance (WS) could fill some of those gaps in surveillance by providing information about disease circulation in areas with limited testing and case reporting, warning of unexpected outbreaks, and helping public health practitioners determine when respiratory virus seasons begin and peak (*5*). WS has been used as a complementary tool alongside COVID-19 clinical surveillance to track trends in SARS-CoV-2 transmission, identify surges or outbreaks, and monitor virus variants (*6**–**13*). When evaluated alongside clinical surveillance data, wastewater data have provided a more complete picture of SARS-CoV-2 transmission in geographic areas with low testing or reporting rates. In addition, evidence suggests that WS might provide an early warning signal for the start of COVID-19 outbreaks (*5**–**7*,*9*,*11**–**13*), which in turn could provide lead time for preparedness measures.

Other viral pathogens, such as influenza A and B viruses, RSV, monkeypox virus, measles virus, human metapneumovirus, and norovirus, have also been successfully detected and quantified in wastewater (*14**–**22*; M.K. Wolfe et al., unpub. data, https://doi.org/10.1101/2022.09.06.22279312). Previous studies have found that wastewater concentrations of influenza virus and RSV correlated with clinical influenza and RSV metrics (*15*). However, evidence for wastewater as an early indicator for influenza A has been inconsistent; some studies found that wastewater concentrations led clinical indicators for influenza (*20*,*21*,*23*), and other studies found that it lagged (*24*). For RSV, multiple studies have found that wastewater lagged RSV clinical data with variable lag periods (*25**–**27*). Those studies support the feasibility of WS for monitoring influenza and RSV transmission in communities and provide insight into the temporal relationship between wastewater concentrations and clinical indicators. However, additional work with longer study periods and a broad variety of comparative clinical datasets could improve the epidemiologic interpretability of wastewater concentrations and help inform the role that WS can play in overall community-level influenza and RSV surveillance. In this study, we examined correlations between influenza A and RSV wastewater concentrations from 18 California sewersheds and data from California’s clinical laboratory–based surveillance systems. We also examined whether wastewater-based surveillance data led or lagged clinical laboratory–based surveillance data.

## Materials and Methods

### Study Period

We evaluated influenza A and RSV surveillance data collected from sentinel laboratory data, mandatory electronic laboratory reporting (ELR) data, and mandatory hospital reporting data during July 3, 2022–March 16, 2024. Our study’s assay for influenza A in wastewater targets the matrix gene, conserved in all influenza A subtypes (e.g., H1, H3, H5) (*28*). We used March 16, 2024, as the cutoff because high H5 virus subtype concentrations have been detected in some California wastewater samples since March 25, 2024. Those high concentrations likely are because of discarded contaminated dairy products or waste from dairy processing facilities, resulting in H5-associated increases in influenza A wastewater concentrations (*29*). To avoid the possibility of nonhuman sources of influenza A in wastewater affecting our correlation analyses, we did not analyze wastewater samples collected after influenza A(H5N1) virus was detected in dairy cattle in California.

### Electronic Influenza A Laboratory Results 

During the study period, all laboratories in California were required to report all PCR-positive influenza inpatient and outpatient laboratory results to local health jurisdictions via an ELR platform. Reporting of influenza A–negative results and RSV results (positive or negative) was not mandatory during the full study period. 

To evaluate correlations between influenza A wastewater and ELR data, we geocoded residential addresses associated with persons who received influenza A virus–positive test results to the spatial boundaries of sewershed catchment areas, as provided by wastewater treatment plant operators (*7*). Each influenza A virus–positive test has an associated specimen date code, which is calculated as the earliest of the following: specimen collection date, specimen received date, and specimen result date. We summed positive tests within each sewershed by using the specimen date code, resulting in daily sewershed-specific ELR counts of influenza A virus–positive specimens. To account for possible day-of-the-week reporting effects while maintaining day-to-day granularity reflecting changes over time, we smoothed daily counts by using a rolling average of positive specimens over the preceding 7 days. Wastewater utilities self-reported population estimates for each sewershed catchment area. Those estimates remained constant over the study period, and we used them to calculate the number of influenza A–positive specimens per 100,000 population.

### Sentinel Laboratory Influenza A and RSV Data

The clinical sentinel laboratory network consists of clinical laboratories throughout California that conduct routine testing as requested by clinicians from inpatient or outpatient settings (*30*). Data reported from those laboratories include the number of positive and negative influenza A and RSV specimens reported to the California Department of Public Health (CDPH) by epidemiologic week (*31*). Sentinel laboratory data are reported by the county where each sentinel laboratory is located, and some test results might represent persons who reside outside the county. During July 3, 2022–March 12, 2023, additional hospital-reported influenza A and RSV test results were available from Santa Clara Valley Medical Center (San Jose, CA, USA) and San Mateo Medical Center (San Mateo, CA, USA), both of which are outside of the sentinel laboratory network. We combined data from those 2 hospitals and from 5 sentinel clinical laboratories in California. We calculated the total number of positive influenza A and RSV samples and percentage test positivity from the hospitals and sentinel laboratories weekly. We calculated percentage positivity for each virus as the sum of positive tests divided by the sum of all tests for that virus. We combined results across all clinical sentinel laboratories and hospitals within each county.

### RSV Testing by Age Group

San Mateo Medical Center and Santa Clara Valley Medical Center provided 2,062 RSV-positive test results by age group for patients 0–4 years, 5–17 years, 18–49 years, 50–64 years, and >65 years of age. We evaluated weekly age-grouped RSV-positive specimen counts; RSV-negative test results were not available by age group for San Mateo Medical Center, so we did not analyze age-grouped test positivity for this analysis.

### Hospital Admission Data

During the COVID-19 public health emergency, all hospitals were mandated to report daily, laboratory-confirmed influenza hospital admissions via the US Department of Human Health and Services Protect/National Hospital Surveillance Network (NHSN) dataset (*32*). Those admission counts did not differentiate between influenza A and B. We aggregated NHSN influenza admission data by the county of the reporting hospital and smoothed by using a rolling sum of the preceding 7 days of daily admissions (i.e., rolling 7-day county-level influenza hospital admissions).

### Wastewater Sample Collection and Processing

The WastewaterSCAN program (https://www.wastewaterscan.org), a collaboration between research partners at Stanford University (Stanford, CA, USA) and Emory University (Atlanta, GA, USA) and laboratory implementing partner Verily Life Sciences (https://verily.com), began monitoring for RSV in November 2021, and for influenza A in January 2022 (*14*). WastewaterSCAN processes wastewater samples using previously described methods (*33*). Wastewater settled solids were collected 3–7 times/week from the primary clarifier of each wastewater treatment plant or from liquid wastewater influent samples using an Imhoff cone (*6*,*33**–**35*); samples were transported to the laboratory at 4^o^C. WastewaterSCAN extracted RNA from settled solids and analyzed viral RNA concentrations by using droplet digital reverse transcription PCR (*33*). We included only WastewaterSCAN data in this study because no other laboratory conducted continuous influenza A and RSV wastewater monitoring in California throughout the full study period. We excluded influenza B from this study because those virus concentrations were only analyzed from a small subset of treatment plants during the full study period.

### Wastewater Data Analysis

We analyzed raw, nonnormalized wastewater concentrations of influenza A and RSV RNA. For wastewater samples without detectable virus, we used a single imputation using half of the laboratory-assigned viral limit of detection. For county-level comparisons, we calculated county wastewater aggregates (Appendix). Analyses included influenza A and RSV wastewater concentrations collected during July 3, 2022–March 16, 2024, from 18 sewersheds in California (Appendix).

### Correlation Analyses

We used Kendall rank correlation to assess correlations between wastewater concentrations and corresponding clinical data because this method is robust to nonnormal and skewed data distributions (*36**–**38*). We defined strong correlations as Kendall τ values >0.49 (*39*) and considered p<0.05 statistically significant. We performed analyses in R version 4.0.4 (The R Project for Statistical Computing, https://www.r-project.org) (Appendix). 

### Lead–Lag Analyses

To assess whether wastewater served as a leading or lagging indicator of circulating viruses, we calculated the cross correlations of wastewater with respect to corresponding clinical laboratory-based datasets for influenza A and RSV. Datasets were compared at daily lag intervals, where wastewater data was offset relative to corresponding clinical data. Date offsets ranged from –35 to 35 days. We calculated the maximum Kendall τ correlation value across all lags and determined the range of lags for which the observed τ was within 0.05 of the maximum τ. When analyzing the range of maximum lagged τ, we inferred that ranges including only negative values indicated wastewater data led clinical laboratory–based data. For ranges including only positive τ values, we inferred that the clinical laboratory–based data led wastewater data. For ranges including both negative and positive τ values, we inferred that neither dataset demonstrated a clear leading signal (Appendix).

### RSV Sensitivity Analysis

We also conducted an exploratory analysis evaluating county-aggregated weekly average RSV wastewater concentrations and weekly age-grouped RSV-positive specimen counts. For that analysis, we repeated Kendall τ correlation and lead–lag analyses by using the number of RSV-positive patient samples per age group reported from the San Mateo and Santa Clara hospitals.

## Results

During the study period, we processed 6,715 wastewater samples from 18 sewersheds for influenza A virus and RSV. A total of 51,847 influenza A virus–positive specimens were reported via ELR and geocoded to included sewersheds. Through clinical sentinel laboratory surveillance, we also included 41,976 influenza A virus–positive specimens from 10 counties and 33,173 RSV-positive specimens from 9 counties. We also included 8,025 influenza hospital admissions from 8 counties reported through NHSN (Table 1).

**Table 1:** Descriptive characteristics for each county and sewershed included in a study of correlations between wastewater concentrations of influenza A and respiratory syncytial viruses and clinical laboratory testing and hospitalization data, California, USA*

County	County-level					Sewershed-level			
	Population	No. influenza hospitalizations	Sentinel laboratory, no. positive specimens			Sewershed	Estimated population	No. ELR influenza A virus–positive specimens	No. wastewater samples processed
			Influenza A	RSV					
Alameda	1,658,061	991	7,184	5,999		East Bay	740,000	4,057	261
Contra Costa	1,158,249	852	6,686	5,167		Central Contra Costa	484,800	3,460	223
						Richmond	100,000	1,215	207
Marin	254,743	144	703	1,544		Novato	53,000	618	241
Sacramento	1,596,281	1,657	6,692	5,092		Sacramento	1,480,000	16,362	548
San Francisco	845,355	574	2,706	2,847		Oceanside	250,000	1,133	524
						Southeast†	750,000	3,607	541
San Mateo	747,777	293	3,503	2,043		Half Moon Bay	28,000	151	224
						San Mateo	150,000	1,149	232
						Silicon Valley	199,000	1,900	546
Santa Clara	1,921,406	3,066	11,336	8,278		Gilroy/Morgan Hill	110,338	1,631	550
						Palo Alto†	236,000	1,225	549
						San Jose	1,500,000	12,746	550
						Sunnyvale	153,000	998	536
Santa Cruz	263,454	NA‡	113	NA‡		Santa Cruz City	70,000	272	238
						Santa Cruz County	94,000	350	239
Sonoma	482,050	448	2,938	1,957		Petaluma	65,000	641	163
Yolo	224,088	NA‡	115	165		Davis	70,717	332	343
Total	9,151,464	8,025	41,976	33,173		Total	6,689,855	51,847	6,715

*ELR, electronic laboratory reporting; NA, not applicable; RSV, respiratory syncytial virus. †The San Francisco Southeast sewershed and the Palo Alto sewershed both encompass small portions of neighboring San Mateo County. As a result, the total population coverage for the 2 San Francisco sewersheds is larger than the San Francisco County population, and the total population coverage for the 4 Santa Clara sewersheds is larger than the Santa Clara County population. ‡Numbers insufficient for inclusion in the analysis.

### Influenza A Correlations

**Figure 1:**
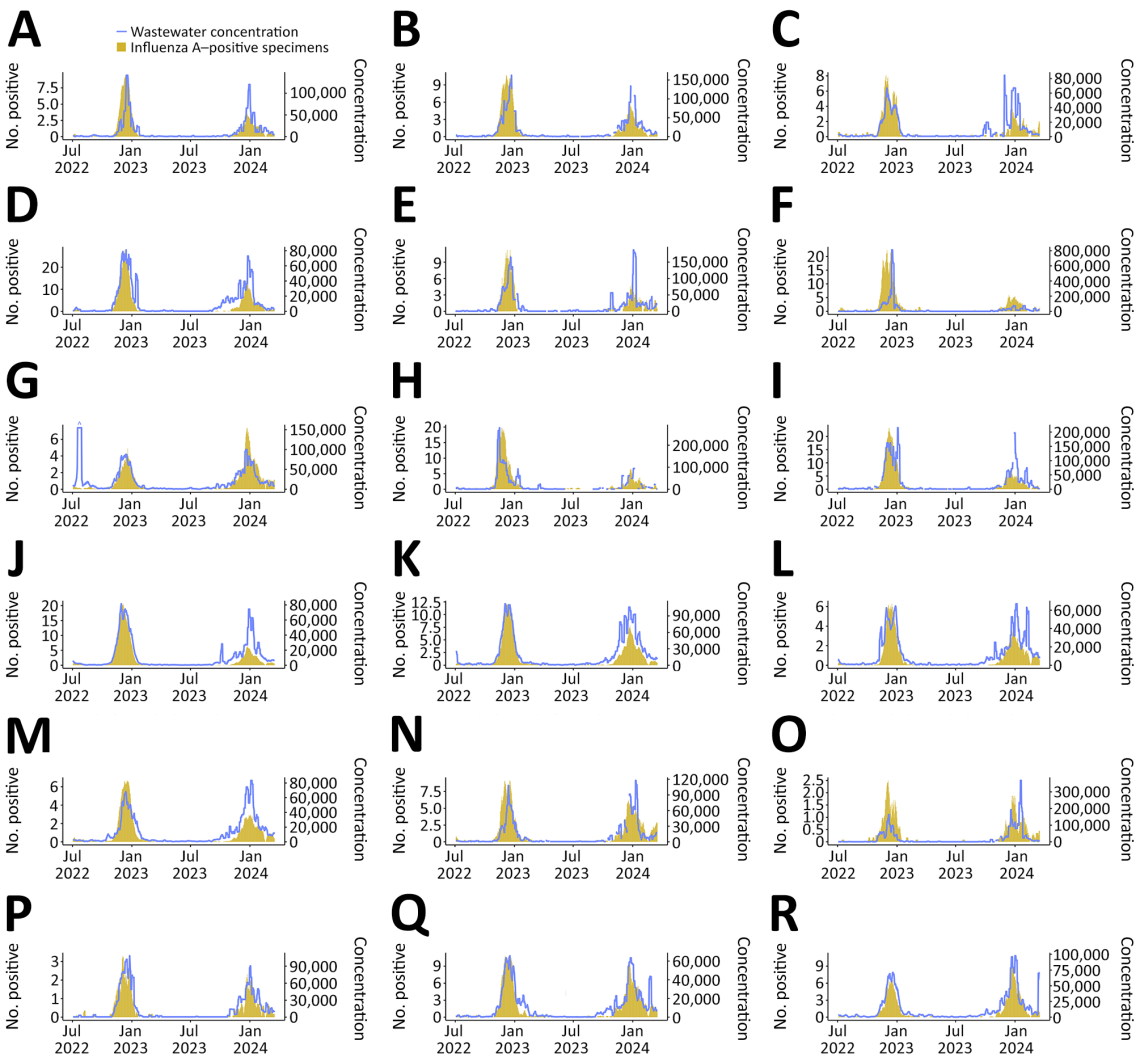
Time series comparison of wastewater and influenza A virus–positive specimens via electronic laboratory reporting from study of correlations between wastewater concentrations of influenza A and respiratory syncytial viruses and clinical laboratory testing and hospitalization data, California, USA, July 2022–March 2024. Reported influenza A virus–positive specimens were geocoded to sewersheds for comparisons. We calculated 7-day rolling average numbers of influenza A virus-positive specimens per 100,000 persons reported via electronic laboratory reporting (yellow bars) and influenza A viral wastewater concentrations in gene copies per gram (light blue line) for 18 sewersheds: A) Alameda (Alameda East Bay); B) Contra Costa (Central Contra Costa); C) Yolo (Davis); D) Santa Clara (Gilroy/Morgan Hill); E) San Mateo (Half Moon Bay); F) Marin (Novato); G) Santa Clara (Palo Alto); H) Sonoma (Petaluma); I) Contra Costa (Richmond); J) Sacramento (Sacramento); K) Santa Clara (San Jose); L) San Francisco (San Francisco Oceanside); M) San Francisco (San Francisco Southeast); N) San Mateo (San Mateo); O) Santa Cruz (Santa Cruz City); P) Santa Cruz (Santa Cruz County); Q) San Mateo (Silicon Valley); R) Santa Clara (Sunnyvale).

We observed strong, positive, and statistically significant correlations between sewershed-level raw wastewater influenza A virus concentrations and the number of ELR influenza A virus–positive specimens per 100,000 persons in the sewershed over the entire study period (median τ 0.64 [range 0.52–0.72]; p<0.001) (Table 2; Figure 1). Of 18 sewersheds assessed for ELR data comparisons, wastewater data led ELR data in 3 sewersheds, ELR data led wastewater data in 3 sewersheds, and neither dataset demonstrated a clear lead in 12 sewersheds (Table 2; Appendix Figure 1).

**Table 2:** Kendall τ correlations between wastewater concentrations of influenza A virus and clinical laboratory testing and hospitalization data, California, USA*

County	County-level correlations											
	Sentinel laboratory % positivity			Sentinel laboratory no. positive specimens			Laboratory-confirmed hospitalizations			Sewershed-level correlations†		
										Sewershed	Population-level	
	Overall	Maximum lag (range)		Overall	Maximum lag (range)		Overall	Maximum lag (range)			Overall	Maximum lag (range)
Alameda	0.56	0.61 (–2 to 15)		0.62	0.66 (–3 to 12)		0.67	0.67 (–14 to 12)		Alameda	0.6	0.61 (–6 to 6)
Contra Costa	0.53	0.6 (2–8)		0.7	0.75 (–9 to 11)		0.65	0.66 (–15 to 5)		Central Contra Costa	0.72	0.73 (1–5)
										Richmond	0.63	0.66 (–11 to –3)
Marin	0.57	0.57 (–10 to 21)		0.61	0.62 (–8 to 16)		0.5	0.53 (–16 to 12)		Novato	0.64	0.65 (–1 to 5)
Sacramento	0.55	0.57 (–10 to 11)		0.69	0.7 (–12 to 10)		0.65	0.66 (–15 to 9)		Sacramento	0.64	0.64 (–7 to 6)
San Francisco	0.57	0.58 (–8 to 14)		0.69	0.69 (–9 to 12)		0.56	0.57 (–15 to 18)		Oceanside	0.6	0.63 (–7 to –3)
										Southeast	0.64	0.65 (–9 to 7)
San Mateo	0.57	0.58 (–9 to 17)		0.65	0.66 (–9 to 17)		0.67	0.67 (–7 to 8)		San Mateo	0.67	0.67 (–2 to 6)
										Half Moon Bay	0.66	0.67 (–3 to 3)
										Silicon Valley	0.62	0.62 (–2 to 5)
Santa Clara	0.47	0.47 (–16 to 17)		0.67	0.67 (–8 to 7)		0.58	0.59 (–12 to 14)		Gilroy/Morgan Hill	0.6	0.6 (–3 to 1)
										San Jose	0.67	0.67 (–5 to 1)
										Sunnyvale	0.7	0.7 (–6 to 2)
										Palo Alto	0.66	0.66 (–1 to 5)
Santa Cruz	0.54	0.61 (1–16)		0.62	0.7 (3–16)		NA	NA		Santa Cruz City	0.7	0.71 (–4 to –1)
										Santa Cruz County	0.75	0.78 (3–9)
Sonoma	0.63	0.69 (2–22)		0.71	0.75 (–3 to 16)		0.64	0.65 (–11 to 6)		Petaluma	0.54	0.57 (3–7)
Yolo	0.57	0.59 (–3 to 19)		0.53	0.55 (–8 to 20)		NA	NA		Davis	0.52	0.53 (–6 to 1)

*Kendall τ correlations between laboratory-confirmed influenza hospital admissions and wastewater influenza A concentrations from ELR, sentinel laboratories, and influenza A–positive specimen counts. Overall correlations represent Kendall τ correlation coefficients for the indicated datasets, calculated over the entire study period with no lags. Maximum lag correlations represent the maximum Kendall τ correlation coefficients for the indicated datasets when comparing lagged clinical datasets to wastewater datasets, including lags of –35 to 35 days. The range of maximum lagged τ (days) represents the range of days around a date of the maximum lagged correlation in which the lagged correlation coefficient is within 0.05 of the maximum τ value. The negative direction indicates that wastewater data is leading clinical data, and the positive direction indicates that wastewater data is lagging clinical data. ELR, electronic laboratory reported. †Data from mandatory ELR reporting calculated as no. positive specimens/100,000 population.

**Figure 2:**
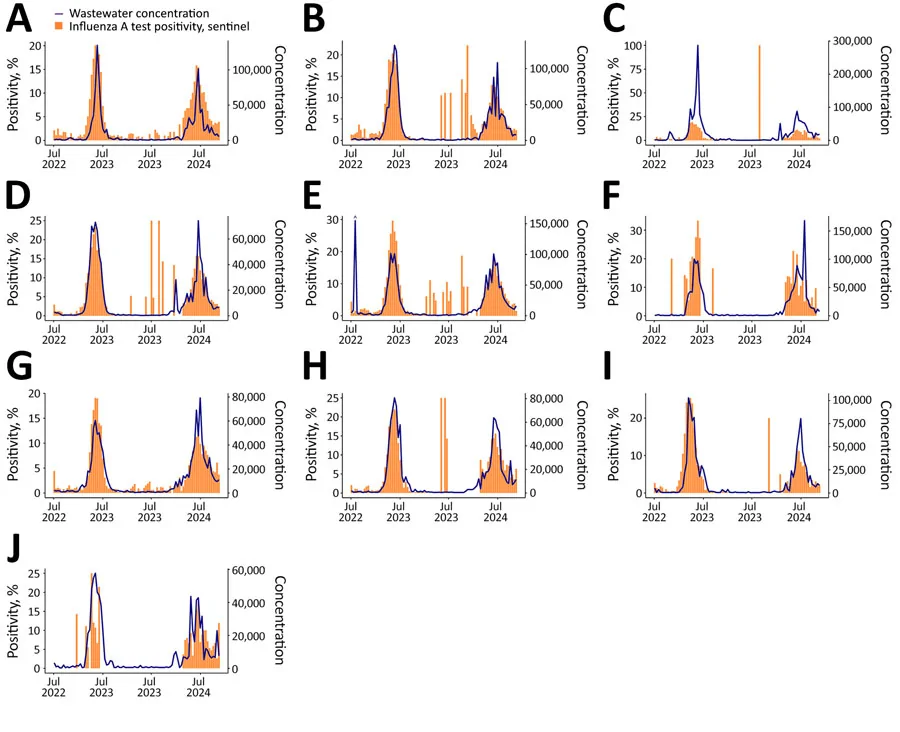
Time series comparison of influenza A virus wastewater concentrations and sentinel laboratory percentage test positivity from study of correlations between wastewater concentrations of influenza A and respiratory syncytial viruses and clinical laboratory testing and hospitalization data, California, USA. Graphs show weekly county influenza A percentage test positivity reported by sentinel laboratories (dark orange bars) and county-aggregated population-weighted averages of nonnormalized influenza A wastewater concentrations in gene copies per gram from all sewersheds sampling within each county (dark blue line) for 10 counties: A) Alameda; B) Contra Costa; C) Marin; D) Sacramento; E) Santa Clara; F) Santa Cruz; G) San Francisco; H) San Mateo; I) Sonoma; J) Yolo.

**Figure 3:**
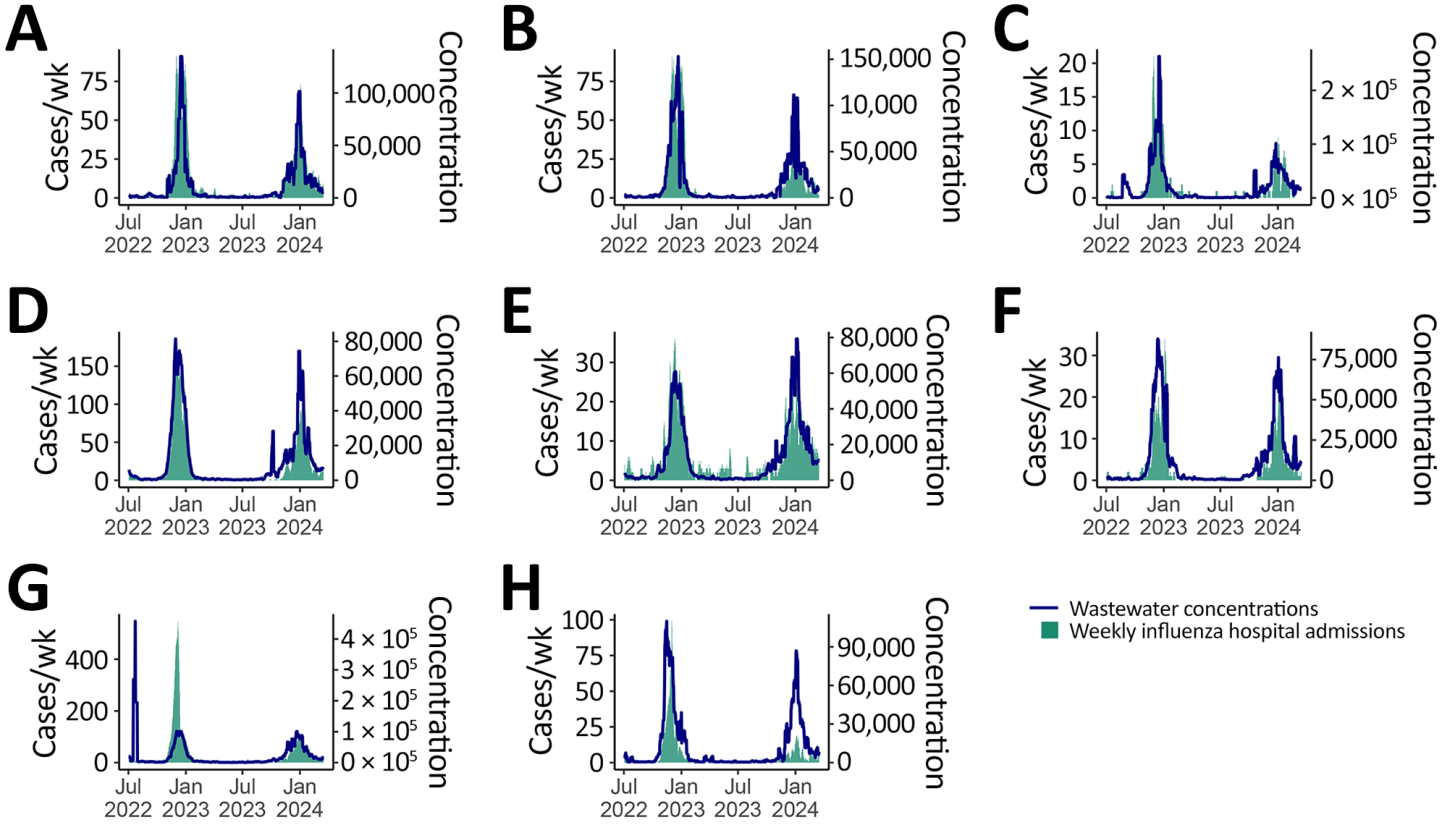
Time series comparison of influenza A virus wastewater concentrations and hospital admissions from study of correlations between wastewater concentrations of influenza A and respiratory syncytial viruses and clinical laboratory testing and hospitalization data, California, USA. Graphs show rolling 7-day county-level influenza hospital admissions (green bars) and county-aggregated population-weighted averages of nonnormalized influenza A wastewater concentrations in gene copies per gram from all sampling sewersheds within each county (dark blue line) for 8 counties: A) Alameda; B) Contra Costa; C) Marin; D) Sacramento; E) San Francisco; F) San Mateo; G) Santa Clara; H) Sonoma.

At the county level, we observed similar results between county-aggregated weekly averages of raw influenza A virus wastewater concentrations and weekly sentinel laboratory influenza A percentage test positivity (median τ 0.57 [range 0.47–0.63]; p<0.001) (Table 2; Figure 2), influenza A virus–positive weekly specimen counts (median τ 0.66 [range 0.53–0.71]; p<0.001) (Table 2; Appendix Figure 2), and rolling 7-day county-level influenza hospital admissions (median τ 0.65 [range 0.50–0.67]; p<0.001) (Table 2; Figure 3). Of 10 counties assessed for sentinel laboratory influenza A comparisons, percentage test positivity led wastewater in 3 counties and neither dataset demonstrated a clear lead in 7 counties. Influenza A virus–positive specimen counts led wastewater in 1 county and neither dataset demonstrated a clear lead in 9 counties. Of 8 counties assessed for influenza hospital admissions, neither wastewater nor hospital admissions demonstrated a clear lead in any county (Table 2; Appendix Figure 3).

### RSV Correlations

**Figure 4:**
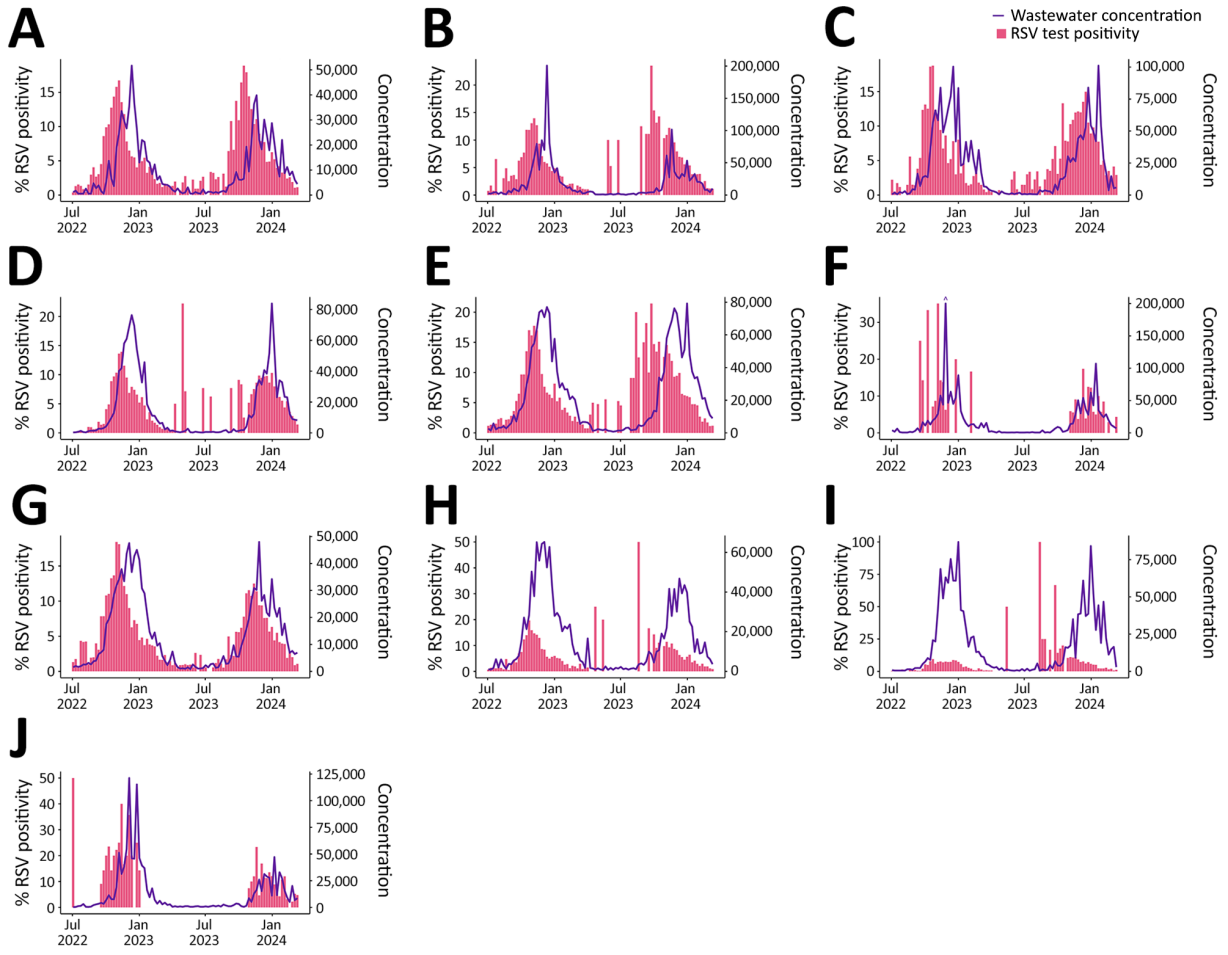
Time series comparison of RSV wastewater concentrations and sentinel percentage test positivity from a study of correlations between wastewater concentrations of influenza A and RSV and clinical laboratory testing and hospitalization data, California, USA. Graphs show weekly county RSV percentage test positivity reported by sentinel laboratories (dark pink bars) and county-aggregated population-weighted averages of nonnormalized RSV wastewater concentrations in gene copies per gram from all sampling sewersheds within each county (purple line) for 10 counties: A) Alameda; B) Contra Costa; C) Marin; D) Sacramento; E) Santa Clara; F) Santa Cruz; G) San Francisco; H) San Mateo; I) Sonoma; J) Yolo. RSV, respiratory syncytial virus.

We observed moderate to strong, positive, and statistically significant correlations in overall comparisons between county-aggregated weekly averages of raw RSV wastewater concentrations and both weekly sentinel laboratory percent test positivity (median τ 0.45 [range 0.40–0.58]; p<0.001) (Table 3; Figure 4) and RSV-positive specimen counts (median τ 0.70 [range 0.48–0.78]; p<0.001) (Table 3; Appendix Figure 4). RSV percentage test positivity led wastewater concentrations in all 9 counties assessed. RSV-positive specimen counts led wastewater in 3 counties and neither dataset demonstrated a clear lead in 6 counties (Table 3).

**Table 3:** Kendall τ correlations between wastewater RSV concentrations sentinel laboratory testing, California*

County	% RSV positivity			No. RSV-positive specimens	
	Overall*	Maximum lag (range)†		Overall*	Maximum lag (range)†
Alameda	0.45	0.65 (16 to >35)		0.64	0.7 (–4 to 18)
Contra Costa	0.46	0.63 (19 to >35)		0.75	0.77 (–2 to 16)
Marin	0.44	0.63 (12 to >35)		0.57	0.65 (2–23)
Sacramento	0.57	0.66 (4 to >35)		0.78	0.82 (–1 to 16)
San Francisco	0.58	0.72 (10 to >35)		0.73	0.75 (–8 to 18)
San Mateo	0.5	0.63 (4–31)		0.7	0.74 (–4 to 10)
Santa Clara	0.42	0.64 (18 to >35)		0.75	0.77 (–5 to 22)
Santa Cruz	0.39	0.48 (1–32)		0.48	0.57 (1–24)
Sonoma	0.4	0.54 (16 to >35)		0.69	0.74 (–2 to 26)
Yolo	0.4	0.64 (28 to >35)		0.48	0.58 (9–34)

*Overall correlations represent Kendall τ correlation coefficients for the indicated datasets, calculated over the entire study period with no lags. Maximum lag correlations represent the maximum Kendall τ correlation coefficients for the indicated datasets when comparing lagged clinical datasets to wastewater datasets, including lags of –35 to 35 days. The range of maximum lagged τ (days) represents the range of days around a date of the maximum lagged correlation in which the lagged correlation coefficient is within 0.05 of the maximum τ value. The negative direction indicates that wastewater data is leading clinical data, and the positive direction indicates that wastewater data is lagging clinical data. RSV, respiratory syncytial virus.

### Age-Specific RSV Correlations

We observed strong, positive, and statistically significant correlations between county-aggregated weekly averages of raw RSV wastewater concentrations and weekly RSV-positive specimen counts among all age groups that had enough positive specimens to calculate correlations (Appendix Table 2). In the 0–4 years of age group, RSV-positive specimen counts led wastewater concentrations by 7–21 days across both counties. In the 5–17 years of age group, RSV-positive specimen counts led wastewater concentrations by 12–31 days in Santa Clara, but we noted no clear lead in San Mateo. Only Santa Clara had enough positive specimens from older age groups to calculate correlations. In age groups >18 years, we noted no clear lead, but the range of maximum lagged τ shifted towards the negative direction, reducing the lead time of positive specimens compared to wastewater (Appendix Table 2, Figure 5).

## Discussion

This study demonstrated that influenza A virus and RSV wastewater concentrations correlated with California clinical laboratory–based surveillance datasets for both viruses. Correlation results were consistent across datasets, including laboratory test results received via ELR, sentinel laboratory–reported data, and laboratory-confirmed influenza hospital admissions. We observed strong and statistically significant correlations for both daily and weekly correlation analyses and at the sewershed and county geographic levels. Influenza A wastewater concentrations did not consistently lead or lag clinical laboratory–based data. RSV wastewater concentrations consistently lagged clinical laboratory–based data. Those findings support the consideration of using WS as a complementary public health tool for monitoring these respiratory viruses.

We found that both clinical laboratory–based and wastewater-based surveillance systems were highly correlated in geographic areas and timeframes where both disease surveillance systems are available. That finding supports the combined, multimodal use of laboratory-based and wastewater-based surveillance for a more complete overall picture of disease transmission. Clinical sentinel laboratory surveillance systems are robust for monitoring respiratory viruses but are subject to biases related to testing rates and case ascertainment. Those biases are particularly notable for mildly symptomatic or asymptomatic cases, within populations with lower testing access and utilization, and for illnesses with readily available home testing. Sentinel laboratory surveillance systems for RSV have historically prioritized surveillance in vulnerable pediatric populations and could underestimate RSV burden in older populations (*40*). WS is also subject to limitations because infections are likely to be missed in diapered children who contribute little waste to sewer systems and because wastewater samples collected at wastewater treatment plants will not represent populations that rely on septic systems (*25**–**27*). 

Strengths of WS include its ability to track changes in community transmission regardless of symptomatology, healthcare utilization, or clinical testing rates. Strategic implementation of WS in locations where clinical laboratory–based surveillance has known gaps, such as rural or medically underserved areas, could serve as a cost-effective way to improve public health surveillance coverage and representation. Of note, small wastewater utilities in rural or underserved areas might not have the resources to easily participate in wastewater monitoring and might require additional financial and logistic support.

This study showed that RSV wastewater data lagged overall clinical laboratory–based data. However, the exploratory age-grouped analyses, although reliant on limited data, suggested that wastewater RSV concentrations were more temporally aligned with RSV-positive specimen counts in age groups >18 years (Appendix Table 2, Figure 5). In the Santa Clara age-grouped RSV-positive specimen counts, RSV was detected in children 0–4 years of age before older age groups, a pattern that another study observed in a larger population and across multiple respiratory virus seasons in 2025 (*41*). Although we did not have sufficient age-specific RSV testing data to draw a conclusion from this exploratory age-group analysis, the RSV wastewater lag observed in this study might be partially explained by early RSV infections among young, diapered children that do not contribute to the wastewater signal. In addition, RSV percent test positivity led wastewater by a larger margin than RSV-positive specimen counts, indicating that percent test positivity could be more sensitive to changes in disease dynamics than both wastewater and case incidence estimates, a dynamic that has also been observed for COVID-19 (*42*).

Although the correlation analyses we used to assess lead–lag patterns did not demonstrate that wastewater provided a leading signal, other wastewater-based data indicators, such as wastewater sample positivity rates, might provide better leading indicators. Another study explored incorporating wastewater sample positivity rates into surge prediction algorithms for COVID-19 (*43*). A similar algorithm could prove useful for providing a leading indicator of the start of influenza and RSV seasons. Also, evaluating correlations and lead–lag patterns during shorter periods within seasons rather than across multiple years might provide more insight into whether wastewater data provides a sensitive or early indicator at timepoints most advantageous to public health, such as the start, peak, and end of each season.

One strength of this study is the inclusion of 20 months of data and multiple clinical laboratory–based surveillance datasets, supporting the robustness of reported results across 2 respiratory virus seasons. This study also implemented a wastewater aggregation method to combine data from multiple sewersheds into county-level wastewater estimates, which enabled more robust comparisons of county wastewater aggregates to county-level clinical surveillance datasets. This analysis focused on data (sewershed and county) at the local level, highlighting that WS can be an effective tool for tracking localized disease transmission.

The first limitation of this study is that we did not normalize raw wastewater data by pepper mild mottle virus (PMMoV), an endogenous human fecal indicator commonly used in WS to normalize viral wastewater concentrations to account for variations in wastewater flow, dilution, and fecal strength (*44*); thus, these study findings might not be directly applicable to surveillance systems that do rely on PMMoV-normalized wastewater concentrations (*44*). A future comparison between raw and PMMoV-normalized data would be valuable, although previous work has shown that PMMoV normalization did not consistently improve correlations between SARS-CoV-2 wastewater concentrations and clinical indicators across all sewersheds evaluated (*44*). Second, the clinical data in this study might have been subject to testing biases, which are challenging to measure and can vary by season and surveillance system. For example, influenza and RSV laboratory testing might have been elevated during the 2022–23 season because of higher demand for testing to identify COVID-19 cases. Thus, correlation estimates reported here might be biased by testing rate variability, potentially reducing the generalizability of these findings to other seasons or geographies. Future analyses should explore how clinical testing rates affect wastewater correlations. In addition, other future analyses could assess correlations and lead–lag patterns when both wastewater concentrations and ELR test results are organized by reporting date, rather than collection date, to clarify wastewater data’s utility for real-time public health response. Third, sentinel laboratory surveillance data and hospital admissions potentially were misclassified to the county where a patient received care, rather than the county of residence. Similarly, wastewater concentrations might represent shedding from residents of neighboring counties who routinely cross sewershed and county boundaries because many of the sewersheds included in this study are in areas with high commuting rates. We also relied on county-aggregated wastewater concentrations, even within counties where WS did not cover an entire county. Given those factors, county-level wastewater and clinical datasets compared in this study likely do not represent identical populations, which could affect the strength of correlations observed between datasets and complicate the comparison of correlations between counties. Nonetheless, demonstrating whether those surveillance systems capture similar disease trends, even in somewhat different populations, is still useful. Fourth, this study only included influenza A wastewater concentrations, but hospital admission sums included both influenza A and B admissions, which could skew the observed temporal relationships observed between wastewater concentrations and hospital admissions in the lead–lag analysis. Future work should investigate correlations between influenza B wastewater concentrations and different clinical surveillance systems. Fifth, this study excluded areas with low numbers of positive specimens counts and hospital admissions, which could limit the generalizability of this analysis to communities with small populations, low testing rates, few cases, or a combination thereof. The strong correlations observed in this study provide encouraging evidence that wastewater could serve as a useful proxy for disease activity in populations with lower testing rates (*45*), but more work is needed for confirmation.

In addition to the future analyses already noted, repeating and expanding analyses with wastewater data aggregated to substate regions, states, multistate regions, and national levels could be useful for determining how those aggregates compare to clinical laboratory data combined across broader geographic levels. In addition, cost-benefit analyses could assess the economic effectiveness of WS as a tool for monitoring influenza and RSV activity and would be useful for jurisdictions considering when and where to implement WS. 

In summary, the results from this study support the use of WS for monitoring influenza A virus and RSV activity. WS could bolster existing surveillance and provide improved public health situational awareness of respiratory virus circulation.

Appendix Additional information on correlations between wastewater concentrations of influenza A and respiratory syncytial viruses and clinical laboratory testing and hospitalization data, California, USA.
