# Supplementary material for: Correlations between Wastewater Concentrations of Influenza A and Respiratory Syncytial Viruses and Clinical Laboratory Testing and Hospitalization Data, California, USA
Source: Emerg Infect Dis. 2026 Aug;32(Suppl 1):S53–65. doi: 10.3201/eid3213.260306 (PMC13585671; doi:10.3201/eid3213.260306)
Supplement: Supplementary file 1 [file 26-0306-Techapp-s1.pdf]

Article DOI: https://doi.org/10.3201/eid3213.260306

EID cannot ensure accessibility for supplementary materials supplied by authors. Readers who have difficulty accessing supplementary content should contact the authors for assistance.

Correlations between Wastewater Concentrations of Influenza A and Respiratory Syncytial Viruses and Clinical Laboratory Testing and Hospitalization Data, California, USA

Appendix

Supplemental Methods

Wastewater Data Analysis: County aggregation

Clinical sentinel laboratory surveillance data and laboratory-confirmed influenza hospital admissions were reported at the county level. Thus, for these correlations, wastewater concentrations from all sewersheds within each county were aggregated. Population-weighted county wastewater aggregates were calculated as described in [Eq. 1], where $S_{ww,i}$ represents rolling weekly averages of wastewater concentrations in each sewershed in the county, Pop_i represents the estimated population of sewershed *i*, and *n* represents the total number of sewersheds with active wastewater monitoring within a given county during a given week. Population estimates were constant throughout the study period and did not reflect temporal fluctuations in population for sewersheds with high commuting or tourism rates.

$$\text{County-level WW} = \frac{\sum_{i=1}^{n}(S_{ww,i} \times Pop_i)}{\sum_{i=1}^{n}(Pop_i)} \text{ [Eq. 1]}$$

Inclusion criteria for sewersheds and counties

Data were restricted to the WastewaterSCAN program due to the program's continuous sampling throughout the study period. For county-level analyses, counties were included if they had continuous wastewater sampling at one or more sewersheds between August 1, 2022 and March 16, 2024 and at least 100 positive influenza A specimens, 100 positive RSV specimens, or

100 influenza hospitalization admissions during the study period for each respective correlation analysis. For sewershed-specific analyses, sewersheds were included if they had continuous wastewater sampling during the same time period and at least 100 ELR influenza A–positive specimens during the study period.

Out of 13 counties with continuous wastewater sampling throughout the study period, these criteria resulted in the exclusion of two counties for the influenza A sentinel surveillance analyses (San Bernardino and San Luis Obispo), three counties for the RSV sentinel surveillance analyses (San Bernardino, San Luis Obispo, and Santa Cruz), and four counties for the influenza hospital admission analysis (San Bernardino, San Luis Obispo, Santa Cruz, and Yolo). Despite having enough positive clinical tests and hospital admissions, Los Angeles was also excluded from all analyses because clinical reporting for this county was separate from the rest of the state during much of the study period. After these exclusions, ten counties were included for the influenza A sentinel laboratory surveillance analyses, nine counties were included for the RSV sentinel surveillance analyses, and eight counties were included for the influenza hospitalization analyses (Table 1). The ten counties included in the sentinel laboratory surveillance analyses included a total of eighteen individual sewersheds with continuous sampling throughout the study period, all of which met the criteria for the influenza A ELR analysis (> 100 positive tests). These criteria were applied because Kendall's correlation analyses are not well-suited for zero-inflated, sparse data, and different analytical methods would be needed for sewersheds and counties with low counts of reported positive specimens.

Correlation Analyses

Because ELR influenza A–positive specimens were geocoded to sewershed boundaries, sewershed-level wastewater influenza A concentrations were used for correlations with ELR data. For correlations with ELR data, for each sewershed, a rolling average of the preceding seven days of daily sewershed-level counts of ELR influenza A positive specimens per 100,000 people was compared to a rolling average of wastewater concentrations from the same preceding seven days. For hospitalization data, rolling sums of all reported influenza hospital admissions in each county in the preceding seven days were compared to county-aggregated rolling averages of wastewater concentrations from the same preceding seven days. Both ELR and hospitalization admission correlations were evaluated on a daily time scale, where each daily data point for ELR positives, hospitalization admissions, and wastewater concentrations represents data from the

preceding seven-day period. For sentinel laboratory surveillance data, weekly percent test positivity and weekly positive specimen counts for influenza A and RSV were compared to weekly averages of county-aggregated wastewater concentrations, by epidemiological week. For sentinel laboratory surveillance correlations, correlations were evaluated on a weekly time scale, resulting in fewer total included data points than the ERL or hospital admission correlation analyses.

Lead/lag analyses

To calculate cross correlations between wastewater surveillance data and clinical data, clinical laboratory–based measures of influenza A and RSV were correlated to wastewater concentrations with date offsets ranging from -35 to +35 days. Influenza ELR data, hospital admission data, and wastewater data were all measured on the daily level and analyzed using rolling averages or sums. For lead/lag analyses with these datasets, wastewater seven-day rolling averages were offset in daily increments in either direction, compared to seven-day rolling averages of ELR data or seven-day rolling sums of hospital admission data.

Sentinel laboratory surveillance data were measured at the weekly level (weekly sentinel laboratory influenza A and RSV percent test positivity and weekly sentinel laboratory influenza A- and RSV-positive specimen counts). To take advantage of the increased temporal resolution of wastewater data when evaluating lead and lag patterns with weekly sentinel laboratory surveillance data, left-aligned rolling averages of wastewater data (i.e. a rolling average of the subsequent seven days of daily county-aggregated wastewater concentrations) were compared to weekly sentinel laboratory surveillance data. This allowed the wastewater seven-day rolling averages to be offset in daily increments up to 35 days in either direction, compared to the weekly sentinel laboratory surveillance data points for each week. A lag of zero represents perfect alignment between the seven-day left-aligned wastewater rolling average (Sunday – Saturday), and the sentinel laboratory surveillance data for the same epidemiological week (also Sunday – Saturday). A lag of -1 represents the seven-day, left-aligned wastewater rolling average from one day earlier (Saturday – Friday), compared to the sentinel laboratory data for the same week (Sunday – Saturday). A lag of +1 represents the seven-day left-aligned wastewater rolling average from one day later (Monday – Sunday), compared to the sentinel laboratory data for the same week (Sunday – Saturday). The date offset of the wastewater rolling averages was done for each week in the dataset, so that each sentinel laboratory weekly value was compared to a seven-

day average of wastewater concentrations preceding that week by -35 days (negative lag) and following that week by +35 days (positive lag).

For interpretation of results, if wastewater were leading sentinel laboratory data, then the strongest correlations would occur with negative lag values. If wastewater were lagging sentinel laboratory data, then the strongest correlations would occur with positive lag values. In either direction, the higher absolute magnitude of the lag value indicates a longer lead time between the leading dataset and the lagging dataset. A table to demonstrate this approach for lags ranging from -3 to 3 days is provided in Appendix Table 1.

Appendix Table 1. Illustration of lead/lag analyses between weekly sentinel laboratory data and rolling averages of county-aggregated wastewater concentrations*

		Day of Week												
Dataset	Lag	Thurs	Fri	Sat	Sun	Mon	Tues	Wed	Thurs	Fri	Sat	Sun	Mon	Tues
Sentinel Lab														
WW	-3													
	-2													
	-1													
	0													
	1													
	2													
	3													

*For each weekly data point in the lead/lag dataset, the ‘Sentinel Lab’ row represents the days captured by the weekly data point for the sentinel laboratory datasets used in these analyses (weekly sentinel laboratory influenza A and RSV percent test positivity and weekly sentinel laboratory influenza A- and RSV-positive specimen counts). The ‘WW’ rows represent the days captured by each weekly data point for the county-aggregated wastewater datasets at each lag step. For each lag, all weekly data points for sentinel laboratory datasets are correlated with county-aggregated wastewater datasets at the indicated day offset.

Appendix Table 2. Kendall's Correlations (τ) between wastewater and weekly age-grouped RSV-positive specimen counts. Kendall's correlation coefficients (τ) and Kendall's lagged correlation coefficients (τ) between RSV wastewater concentrations and weekly age-grouped RSV-positive specimen counts, reported by two different hospitals. For each age group, weekly averages of population-weighted county wastewater aggregates of raw RSV wastewater concentrations from all sewersheds sampling within each county were compared to weekly RSV-positive specimen counts

County	Age group, y	No. RSV-positive specimens	Overall τ*	Max lagged τ†	Range of maximum lagged τ (days)‡
Santa Clara Valley Medical	All ages combined	1,912	0.77	0.87	7 to 17
	0–4	1,034	0.73	0.86	7 to 19
	5–17	433	0.64	0.79	12 to 31
	18–-49	217	0.76	0.76	-4 to 1
	50–64	119	0.79	0.79	-18 to 3
	>65	109	0.68	0.71	-18 to 8
San Mateo Medical Center	All ages combined	150	0.69	0.8	13 to 21
	0–4	128	0.64	0.76	14 to 16
	5–17	19	0.51	0.57	-1 to 22
	18–-49	1	NA	NA	NA
	50–64	0	NA	NA	NA
	>65	2	NA	NA	NA

*Overall correlations (τ) represent Kendall's correlation coefficients for the indicated datasets, calculated over the entire study period with no lags.
†Max lagged correlations (τ) represent the maximum Kendall's correlation coefficients for the indicated datasets when comparing lagged clinical datasets to wastewater datasets, including lags of -35 to +35 days.
‡The range of maximum lagged τ (days) represents the range of days around a date of the maximum lagged correlation in which the lagged correlation coefficient is within 0.05 of the maximum τ value. The negative direction indicates that wastewater data is leading clinical data, and the positive direction indicates that wastewater data is lagging clinical data.

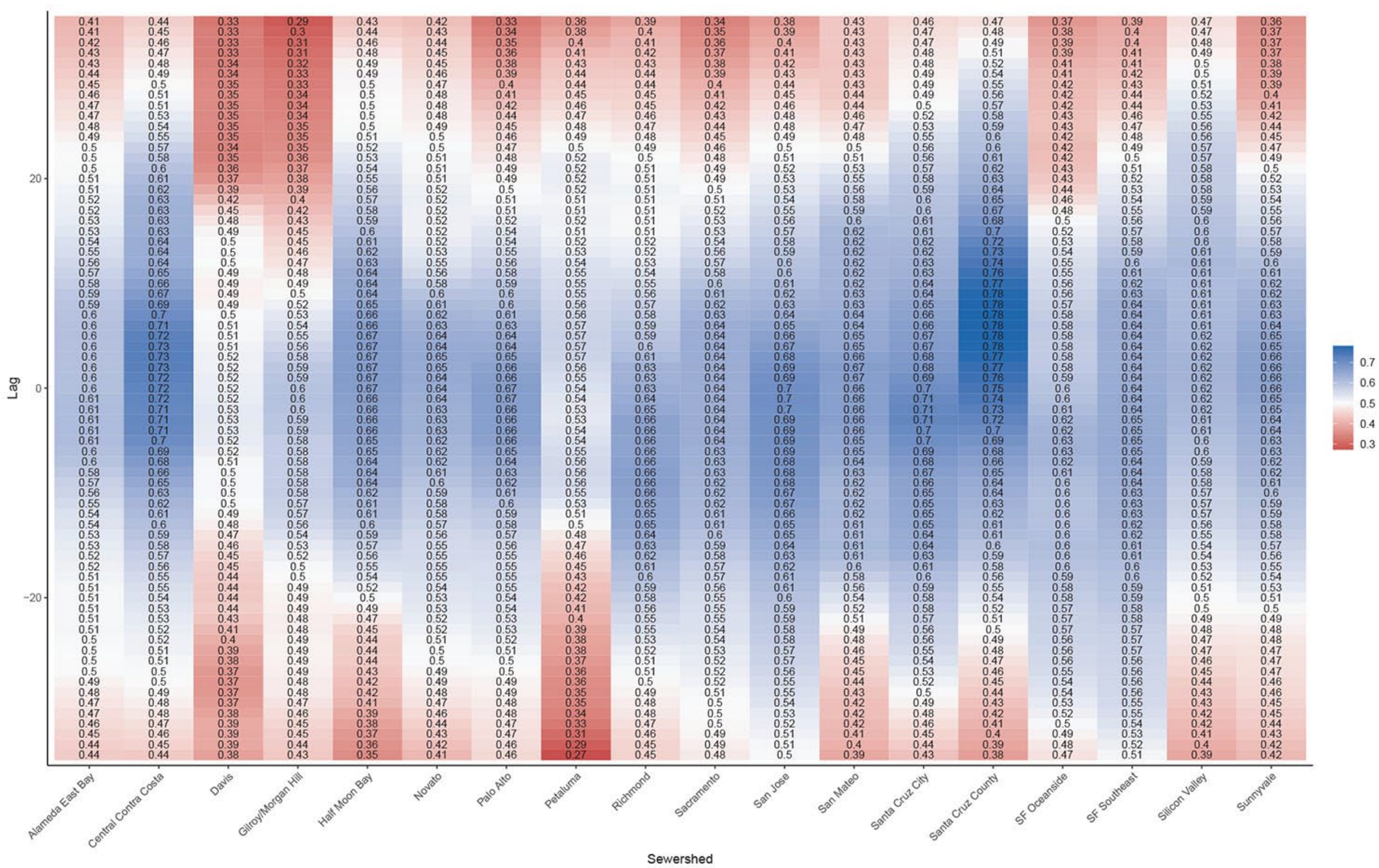

Appendix Figure 1. Lead/lag analysis of sewershed-level influenza A wastewater concentrations and sewershed-level ELR-reported influenza A positive specimens. Rolling 7-day averages of daily sewershed-level ELR-reported influenza A positive specimens/100,000 people were compared to rolling 7-day averages of raw wastewater concentrations. Wastewater data was displaced by one day

increments, relative to influenza A positives, in both directions, and Kendall's correlations were analyzed at each increment. Leading and lagging correlations were calculated between wastewater data displacements of -35 days to + 35 days (where the negative direction indicates that wastewater data is leading influenza A–positive specimen counts and the positive direction indicates that wastewater data is lagging influenza A–positive specimen counts.)

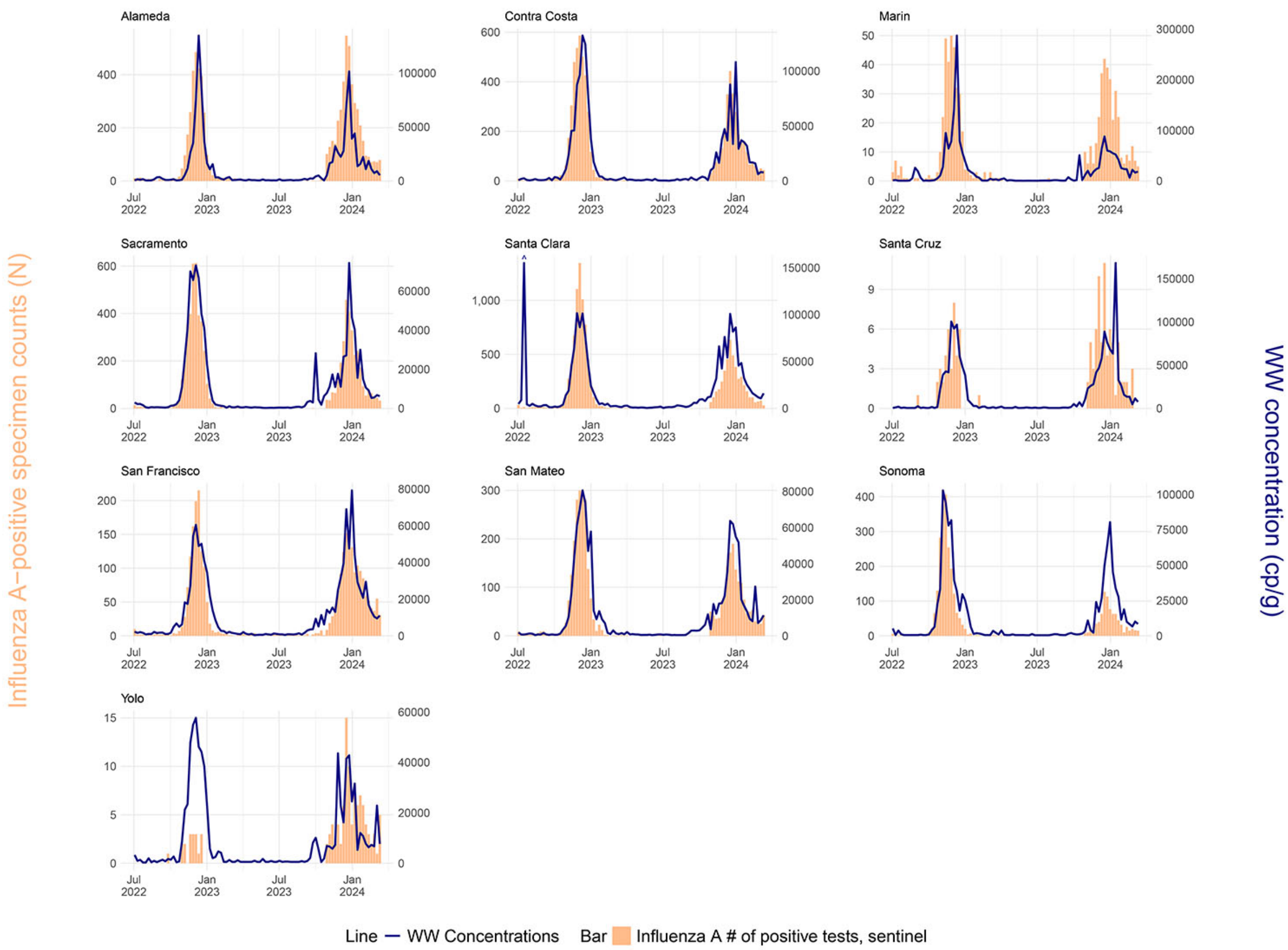

Appendix Figure 2. Time series comparison of county-aggregated influenza A wastewater concentrations and weekly influenza A–positive specimen counts reported through sentinel laboratory surveillance. Weekly averages of population-weighted county wastewater aggregates of raw influenza A wastewater concentrations from all sewersheds sampling within each county (dark blue line) and weekly influenza A–positive specimen counts reported by sentinel laboratories (light orange bars).

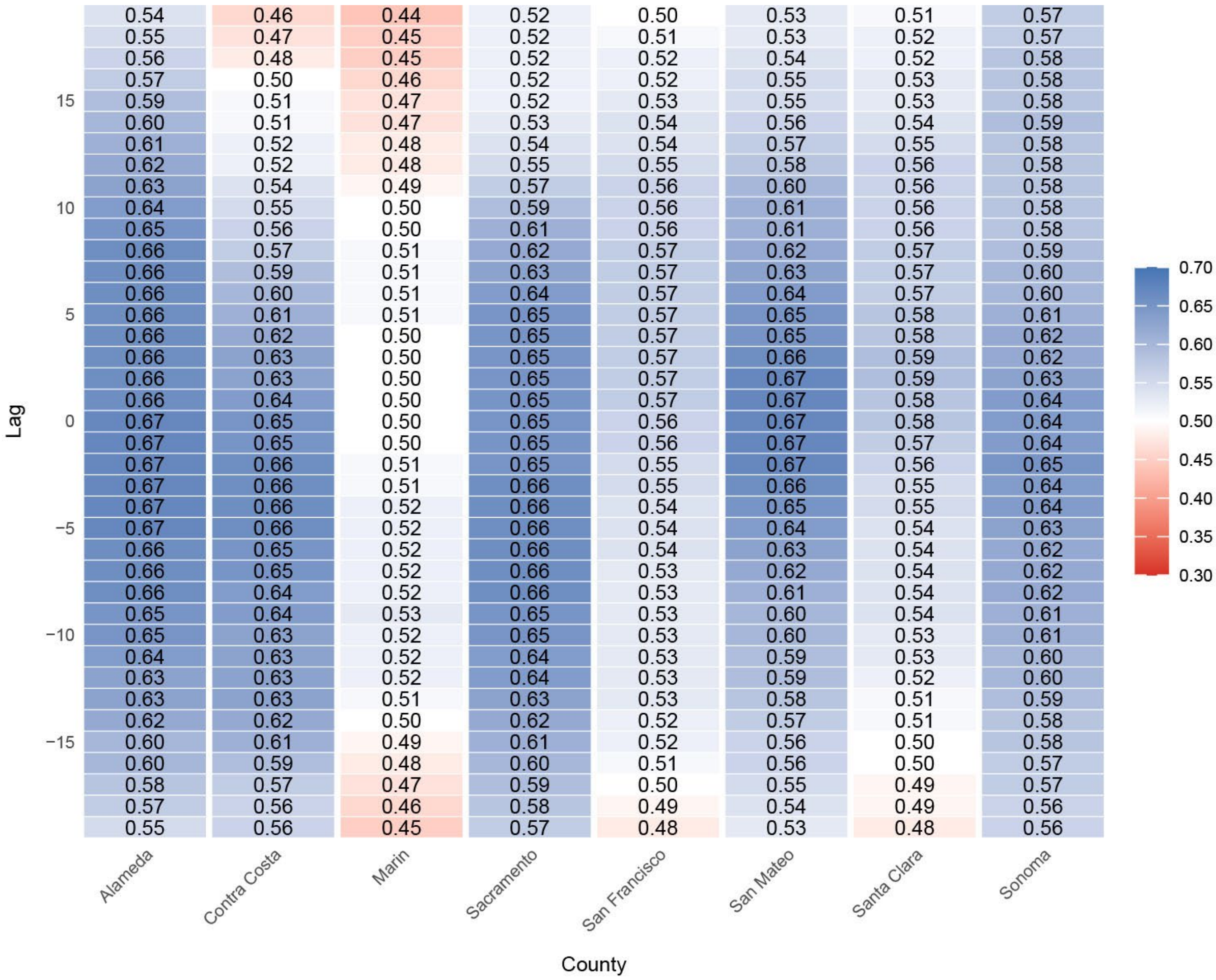

Appendix Figure 3. Lead/lag analysis of county-aggregated influenza A wastewater concentrations and rolling seven-day county-level influenza hospital admissions. Rolling right-aligned seven-day sums of daily county-level laboratory-confirmed hospital admissions compared to population-weighted sums of 7-day averages of raw influenza A wastewater concentrations from all sewersheds sampling within each county. Wastewater data was displaced by one-day increments, relative to hospital admissions, in both directions, and Kendall's correlations were analyzed at each increment. Leading and lagging correlations were evaluated for displacements of up to -35 days and + 35 days (where the negative direction indicates that wastewater data is leading hospital data and the positive direction indicates that wastewater data is lagging hospital data). Plot only shows displacements up to -20 and +20 days to improve plot readability.

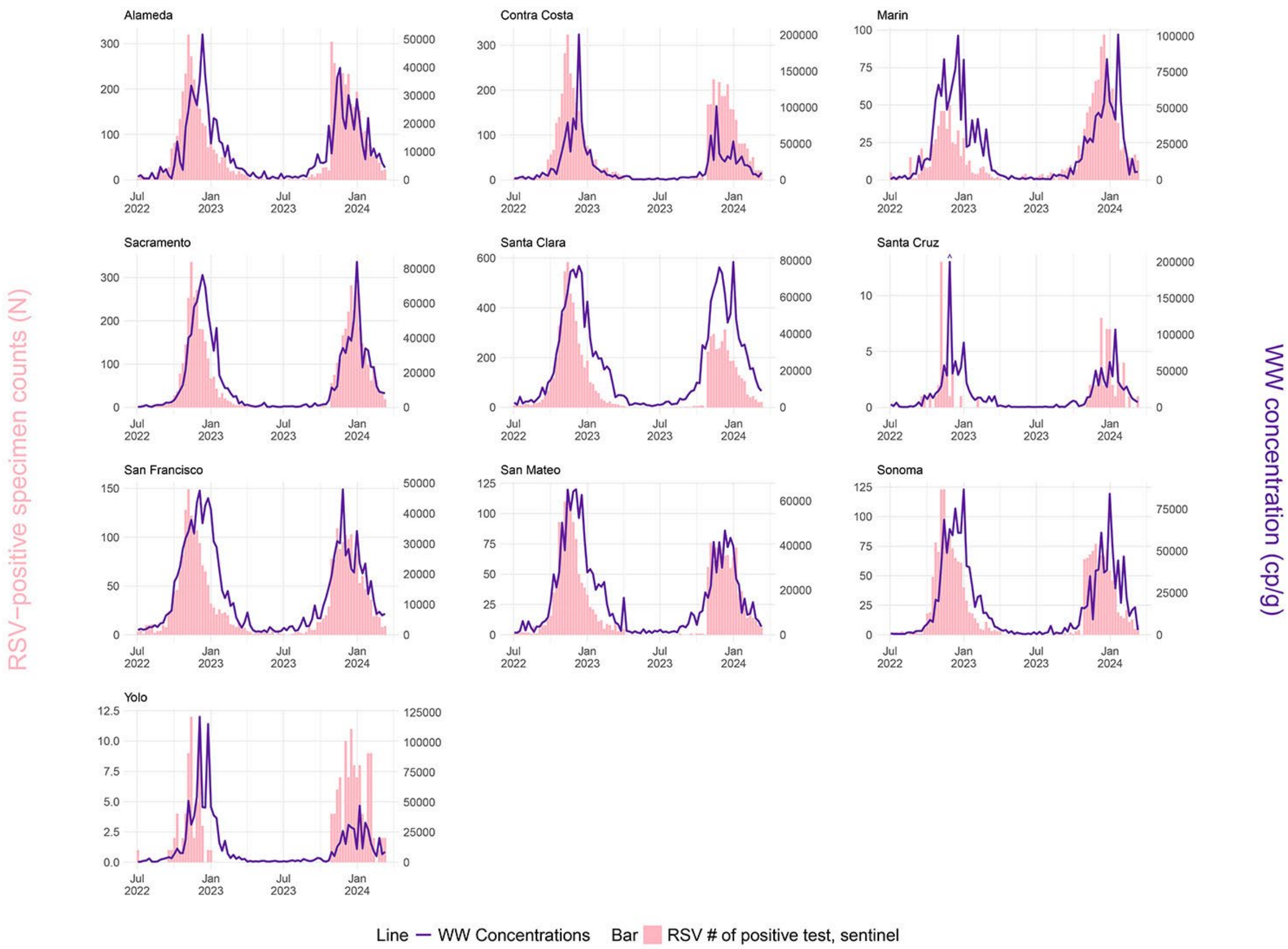

Appendix Figure 4. Time series comparison of county-aggregated RSV wastewater concentrations and weekly RSV-positive specimen counts reported through sentinel laboratory surveillance. Weekly averages of population-weighted county wastewater aggregates of raw RSV wastewater concentrations from all sewersheds sampling within each county (dark purple line) and weekly influenza A–positive specimen counts reported by sentinel laboratories (light pink bars).

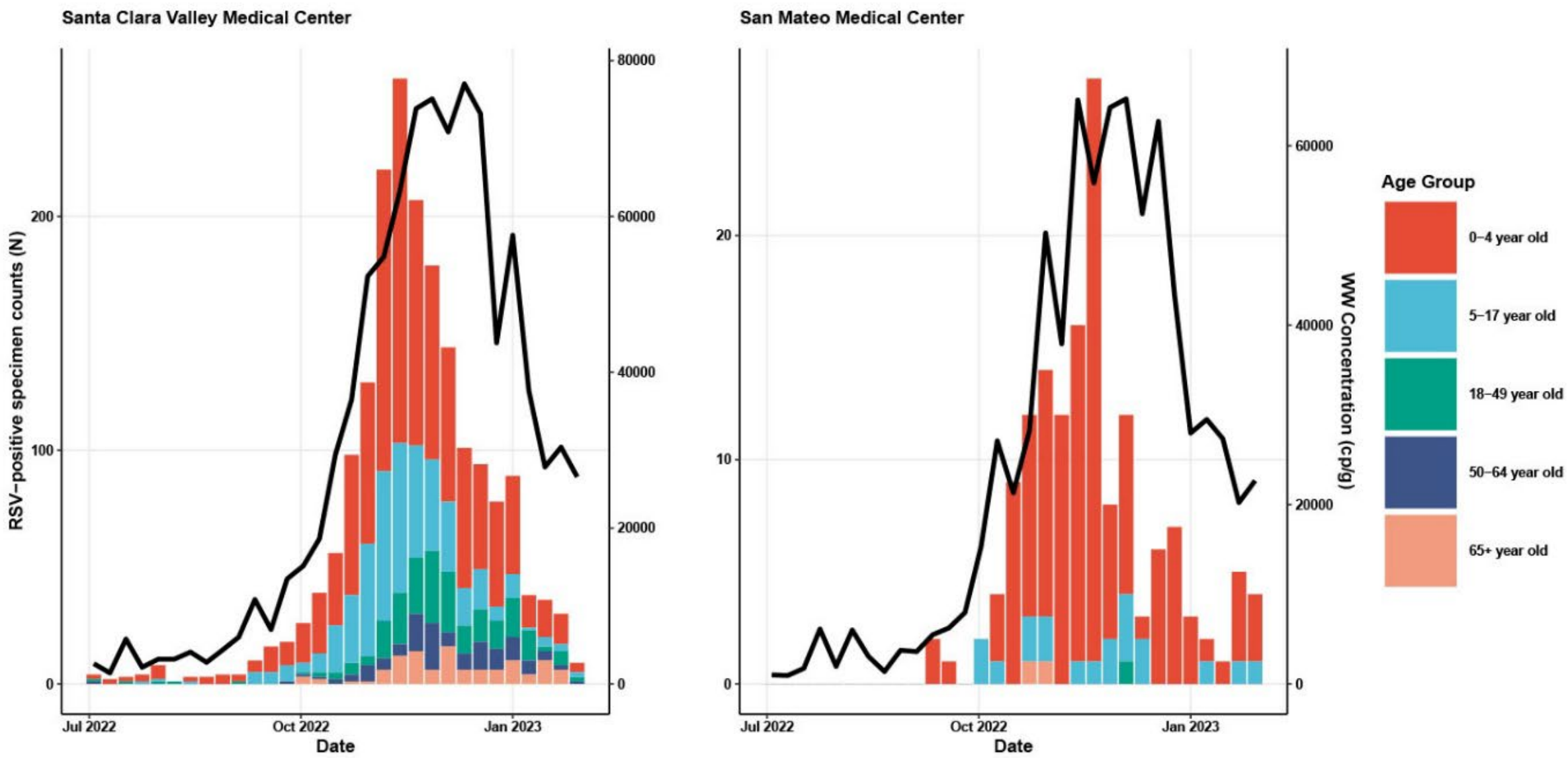

Appendix Figure 5. Time series comparison of county-aggregated RSV wastewater concentrations (black line) and hospital-reported weekly age-grouped RSV-positive specimen counts (color-coded bars).
